# Research on college students’ mental health education: the mediating role and the moderating effect of resource allocation

**DOI:** 10.3389/fpsyg.2025.1687722

**Published:** 2026-01-12

**Authors:** Fawei Li, Hui Yao

**Affiliations:** Lianyungang Normal University, Lianyungang, China

**Keywords:** college students, dynamic monitoring, mental health education, precise intervention, resource allocation

## Abstract

Amid global digital transformation and the expansion of higher education, college students are confronting increasingly salient mental health challenges stemming from academic pressure, career uncertainty, and social adaptation difficulties—rendering the development of a systematic mental health education system for college students an indispensable core strategy to mitigate the global college student mental health crisis. While existing research largely centers on fragmented intervention strategies or problem delineation, it lacks a systematic examination of the core components and intrinsic logic underlying a multi-level, multi-stakeholder collaborative support system. To address these research gaps, this study leverages multidimensional synergy theory to develop a tailored mental health education support system for college students, while clarifying the mediating role of educators’ mental health literacy and the moderating effect of mental health resource allocation. This system encompasses six core modules: educators’ mental health capacity building; dynamic monitoring of students’ psychological states; development of grassroots psychological service teams; tiered psychological crisis intervention; co-construction of mental health-conducive classroom environments; and targeted support for special student groups. Looking ahead, future research should transcend single-module effectiveness testing and shift toward cross-module, cross-level empirical validation of the mediating mechanism of educators’ mental health literacy and the moderating effect of mental health resource allocation within the system—thereby advancing a paradigm shift in the field from fragmented interventions to systematic science.

## 1 Introduction

### 1.1 Research background

Amid global digital transformation and the rapid expansion of higher education, college students worldwide face increasingly complex psychological challenges stemming from academic pressure, career uncertainty, and social adjustment difficulties (Shek et al., 2023). This is not a China-specific issue but a pervasive concern within the global higher education landscape. While nearly one billion people globally live with diagnosable mental health conditions (World Health Organization, 2022), these disorders remain widely stigmatized and misunderstood—with access to care severely constrained by critical shortages of mental health services and resources.

Regionally, WHO data underscores the global reach of mental health disorders, with prevalence rates ranging from 10.9% in Africa, 11.7% in the Western Pacific Region, and 13.2% in Southeast Asia to 14.2% in Europe and 15.6% in the United States (World Health Organization, 2022). This epidemiological pattern highlights the urgent need for global action on mental health. In China’s higher education context, however, college student mental health issues are not only highly prevalent but also marked by substantial regional disparities. Compounding this challenge is the long-standing bias in traditional evaluation systems that prioritize academic achievement over mental health—further amplifying students’ psychological vulnerability (Chen et al., 2024; Sun et al., 2024; Yang et al., 2023). A national survey of Chinese undergraduates revealed that 17.8% report mental health difficulties of varying severity (Wu et al., 2021). Collectively, these findings underscore the imperative of prioritizing college student mental health and optimizing existing mental health education frameworks—especially amid persistent global shortages of mental health resources.

### 1.2 Core mental health dilemmas of college students

College students face multiple interconnected psychological challenges during their campus years, which can be categorized into four primary dimensions:

#### 1.2.1 Adaptation problems: triple challenges from family to campus

When college students transition from familiar family environments to unfamiliar campuses, they face multiple adaptation challenges related to living environments, learning patterns, and interpersonal relationships. In terms of living environments, geographical differences often lead to discomfort with climate and diet. For example, southern students studying in the north frequently struggle to adapt to the dry climate and a diet dominated by wheat-based foods. Such significant changes in living conditions can cause physical discomfort, which in turn affects mental states and triggers negative emotions such as irritability and anxiety (Asif et al., 2020). This is a stress response to a new environment; if not adjusted promptly, it may lead to more severe psychological problems.

In terms of learning patterns, universities place significantly higher demands on self-directed learning. Some students fail to adjust their learning strategies in a timely manner, leading to declining academic performance and subsequent negative emotions like anxiety and inferiority. University courses are rich in content and highly specialized, requiring students to possess strong self-directed learning and time management skills (Fu et al., 2025). However, some students who were accustomed to strict supervision and passive learning modes in high school struggle to adapt to university life, resulting in low learning efficiency, unsatisfactory grades, and a gradual loss of self-confidence (Awadalla et al., 2020).

In interpersonal relationships, college students need to rebuild their social networks. Introverted students or those with weak social skills often encounter conflicts with roommates due to differences in living habits and personalities, experiencing loneliness and a sense of isolation. The quality of roommate relationships directly impacts students’ mental health (Quinn et al., 2023). For instance, students with poor living habits (e.g., neglecting personal hygiene) may disrupt others’ quality of life and trigger conflicts; introverted students may struggle to communicate actively with others, finding it difficult to integrate into the group, which over time leads to loneliness. If these interpersonal problems are resolved timely and properly, they can have a positive impact on students’ mental health.

#### 1.2.2 Emotional problems: the intertwined dilemma of stress and anxiety

Anxiety is a common emotional issue among college students. Factors such as academic pressure, employment competition, and career planning can all trigger anxiety. For example, students worry about poor grades around exams, feel confused about the future during job hunting, or need to participate in various exams—keeping them in a state of prolonged tension and anxiety (Chen et al., 2024). Prolonged exposure to stress and negative emotions facilitates the emergence of depressive feelings, which not only affect students’ learning and daily lives but may also harm their physical health. For instance, some students experience loss of appetite, decreased immunity, and other issues due to long-term depression, severely impacting their normal study and daily functioning.

Furthermore, college students often exhibit significant emotional fluctuations. When facing interpersonal conflicts or stressful situations, they may struggle to control anger effectively and act impulsively. As college students are in a stage of incomplete psychological development, their emotional regulation abilities are relatively weak, making them prone to emotional outbursts and irrational behaviors when encountering setbacks or conflicts—causing harm to themselves and others (Öztekin et al., 2025).

#### 1.2.3 Self-cognition issues: manifestations of conceit, inferiority, and confusion

College students frequently encounter self-cognition challenges such as excessive conceit, inferiority complexes, and self-cognitive confusion (Rafiq and Linden, 2024). Some develop conceit upon entering university, relying on their strong academic performance in high school or specific specialized skills. They overestimate their abilities, belittle others, and act dictatorially in team collaborations, refusing to accept others’ opinions. For example, in group assignments, conceited students often believe their ideas are the most valid and impose their views on team members, leading to frequent conflicts. Such excessive conceit hinders their ability to integrate into the collective. When facing setbacks, they experience a significant psychological gap, making them prone to negative emotions such as anxiety and anger.

In contrast to conceit, many students struggle with inferiority for various reasons. Some from economically disadvantaged families feel inferior when seeing classmates wearing brand-name clothing or using high-end electronic devices. Others lack confidence in interpersonal communication due to concerns about their appearance or body image, fearing to initiate interactions. Under the higher academic requirements and fierce competition in university, some doubt their learning abilities due to unsatisfactory grades. Prolonged inferiority can lead to low mood, loneliness, and introversion, severely impacting mental health and daily academic functioning (Kotera et al., 2022). University is a diverse environment where students face pressures and choices related to studies, socialization, and career planning. Some develop self-cognitive confusion, unaware of their hobbies, strengths, or weaknesses, and feel uncertain about the future. When selecting specialized courses or club activities, they often follow trends blindly—joining popular clubs or courses without reflection—only to find they are ill-suited, thus falling into confusion and anxiety.

#### 1.2.4 Internet dependency: the psychological gap between virtual and real worlds

The widespread adoption of the Internet has led some college students to overindulge in online activities. Prolonged engagement in online gaming and social media not only impairs academic performance but also causes physical fatigue, vision deterioration, and social dysfunction. The Internet’s virtuality, entertainment value, richness, and convenience easily attract students (Rahul et al., 2025). Some students addicted to online gaming and social media spend excessive time online, reducing their study hours and leading to declining grades. Meanwhile, prolonged Internet use undermines physical health, such as causing fatigue and myopia.

Excessive reliance on virtual socialization weakens real-life interpersonal competence. The gap between the virtual and real worlds further impacts mental health. The convenience and anonymity of virtual socialization allow students to communicate easily online, but they struggle with face-to-face interactions, leading to social barriers. The significant disparity between virtual experiences and real life triggers dissatisfaction with reality and a desire to escape, undermining mental well-being.

### 1.3 Research questions and hypotheses

#### 1.3.1 Research questions

Given the complexity and urgency of mental health issues among college students worldwide, this study, based on existing literature related to mental health, focuses on the following key research questions:

*RQ1:* How to break through the limitations of existing single-dimensional intervention models and construct a comprehensive support system for college student mental health education that integrates the functions of prevention, monitoring, intervention, and development, while encompassing multiple stakeholders and stages?

*RQ2:* How does educators’ mental health literacy exert a mediating role in this support system to realize the transformation pathway from system resource input to improvements in students’ mental health?

*RQ3:* How to design targeted and operable support mechanisms tailored to the differentiated needs of special student groups (e.g., those facing financial hardship, academic setbacks, or mental health challenges) and address the gap of insufficient attention to vulnerable groups?

#### 1.3.2 Research hypotheses

Based on the aforementioned research questions, this study proposes the following research hypotheses:

*H1:* Compared with conventional mental health education models, the implementation of a mental health education system integrating six core modules—educator capacity building, dynamic monitoring, grassroots team development, tiered intervention, co-construction of classroom environments, and targeted support for special student groups—will significantly improve college students’ overall mental health (e.g., psychological resilience, emotional stability, life satisfaction) and academic adaptability (e.g., academic engagement, academic accomplishment).

*H2:* Educators’ mental health literacy plays a mediating role between the implementation of the mental health education system and improvements in students’ mental health. Specifically, the implementation of the system will enhance educators’ mental health literacy, which in turn promotes improvements in students’ mental health; after controlling for this mediating pathway, the direct effect of the system implementation on students’ mental health will weaken or become non-significant.

*H3:* Mental health resource allocation plays a moderating role both in the relationship between system implementation and educators’ literacy, and in the effectiveness of the system itself. Specifically: the higher the level of resource allocation (e.g., professional staff-to-student ratio, funding investment, platform construction), the stronger the promoting effect of system implementation on educators’ mental health literacy; simultaneously, the higher the level of resource allocation, the higher the degree of implementation, professional depth, and functional effectiveness of each module.

*H4:* There is a synergistic effect among the core modules within the system. Specifically: (1) Grassroots team development (Module 3) will effectively expand the coverage of dynamic monitoring (Module 2) and improve its early warning accuracy; (2) Co-construction of classroom environments (Module 5) will reduce the incidence of psychological crisis events, thereby alleviating the workload of tiered intervention (Module 4); (3) The successful practice of targeted support for special student groups (Module 6) will positively feedback and enhance the inclusiveness and supportiveness of classroom environments (Module 5).

### 1.4 Research purpose and significance

This study adheres to the principles of “prevention first, integration of prevention and intervention, and targeted intervention,” aiming to construct a comprehensive psychological support system for college students, clarify the mediating role of educator mental health literacy and the moderating effect of resource allocation in the system, and provide theoretical and practical guidance for universities to build a mental health education ecosystem in line with the “all-staff, whole-process, all-dimensional” educational paradigm. Theoretically, it enriches the research on systematic intervention of college students’ mental health; practically, it provides actionable strategies for university administrators, educators, and mental health practitioners to improve the quality of mental health services.

## 2 Methods

### 2.1 Literature search strategy

The core themes of this literature search are “college student mental health” and “mental health influencing factors in college students,” with retrieval conducted across multidisciplinary databases including Scopus, PubMed, Web of Science, CNKI, and the WHO official website; The search scope has been updated to include Chinese and English literatures published as of December 16, 2025, including online-first published papers. Combined subject terms and free-text words are used as search terms: English terms include “college student mental health,” “mental health influencing factors,” “multidimensional collaboration in mental health education,” “mental health resource allocation,” and “educator mental health literacy,” while Chinese terms include “College students’ mental health,” “Factors affecting mental health,” “Multidimensional collaboration in mental health education,” “Mental health resource allocation,” and “Mental health literacy of educators”; the search formula is defined as (“college student mental health”) AND (“mental health influencing factors” OR “mental health impact factors”) AND (“multidimensional collaboration in mental health education” OR “mental health education multidimensional collaboration”) AND (“mental health resource allocation”) OR (“educator mental health literacy”).

### 2.2 Inclusion and exclusion criteria

Inclusion Criteria: Research objects are college students (undergraduates, postgraduates); Research content focuses on college students’ mental health issues, influencing factors, or mental health education intervention models; Research design is empirical (cross-sectional study, longitudinal study, intervention study) or systematic review/meta-analysis; Literature provides complete data or clear research conclusions.

Exclusion Criteria: Research objects are non-college student groups (e.g., middle school students, social workers); Research content is unrelated to college students’ mental health (e.g., mental health of community residents); Literature type is conference abstract, book review, or theoretical discussion without empirical support; Data is incomplete, conclusions are ambiguous, or the quality of literature is too low to meet the evaluation standards.

### 2.3 Literature screening and quality assessment

Screening Process: First, two researchers independently screened literature titles and abstracts to exclude irrelevant literature; then, read the full text of the remaining literature for secondary screening to determine the final included literature; in case of disputes, resolved through discussion with a third researcher.

### 2.4 Analysis and synthesis

This study integrates both thematic analysis and narrative synthesis methodologies to systematically address the research objective. Initially, the research focus is subjected to descriptive categorization to establish a structured analytical foundation. The core analytical process is characterized by proactive integration and theoretical construction: rather than presenting findings in a passive, enumerative manner, we adopt a problem-driven approach centered on “how to develop a logically coherent and comprehensive support system.” From the included literature, we extract relevant theoretical underpinnings, practical components, and inherent logical linkages, which are then subjected to systematic synthesis, deductive reasoning, and rigorous articulation to formulate the six-module framework detailed in subsequent sections. Notably, this integrative and constructive analytical process itself constitutes the primary theoretical contribution of the present review.

## 3 Theoretical framework and core concept definition

### 3.1 Core theoretical basis

#### 3.1.1 Multidimensional collaboration theory

The theory emphasizes that the solution to complex public problems requires the joint participation and coordinated cooperation of multiple subjects. In college students’ mental health education, it is reflected in the linkage of universities, educators, students, and families, and the integration of resources from all parties to form a collaborative governance pattern of mental health services. This theory provides a basis for the construction of grassroots psychological service teams and cross-departmental collaboration in crisis intervention in this study.

#### 3.1.2 “All-staff, whole-process, all-dimensional” educational paradigm

This paradigm requires that mental health education cover all educators and students, run through the entire process of college students’ campus life, and involve all aspects of teaching, management, and campus culture, realizing the full coverage and deep integration of mental health education. It provides a guiding framework for the overall design of the six core modules of the support system in this study.

### 3.2 Core concept definition

#### 3.2.1 Educator mental health literacy

It refers to the knowledge reserves, cognitive level, and practical ability of university teachers, counselors, and administrative staff in identifying mental health problems, providing preliminary psychological guidance, and regulating their own mental state. It is the core mediating variable affecting the effectiveness of student mental health education.

#### 3.2.2 Mental health resource allocation

It mainly includes the quantity of professional mental health personnel, the investment of service funds, and the construction of service platforms in universities. It plays a moderating role in the operation of the psychological support system, and reasonable resource allocation can significantly enhance the system’s service efficiency.

#### 3.2.3 Hierarchical crisis intervention

It refers to the classification and disposal of students’ psychological crises according to their severity, and the formulation of targeted intervention strategies for different levels of crises, so as to realize the precise response to psychological emergencies.

#### 3.2.4 Special student groups

Special student groups in universities, including students from low-income families, students with mental health conditions, academically challenged students, and students with disabilities.

#### 3.2.5 Student party members

Student Party member is the student leader with a strong sense of responsibility and organization and coordination ability, who has special advantages in contacting, uniting and leading college students. Under China’s institutional context, these peer leaders are formally affiliated with political organizations, whereas in other contexts, they may be residential advisors, student association presidents, academic mentors, among others.

### 3.3 Theoretical application logic

Multidimensional Collaboration Theory guides the multi-subject participation mechanism in the support system: universities are responsible for resource allocation and system construction, educators play a core role in intervention implementation, students participate in peer support and self-regulation, and families provide emotional backing, forming a collaborative governance pattern. The “All-Staff, Whole-Process, All-Dimensional” Educational Paradigm ensures the comprehensiveness of the support system: covering all educators and students through educator capacity building and special group support; running through the entire campus life cycle through dynamic monitoring and hierarchical intervention; involving teaching, management, and campus culture through class environment co-construction and grassroots team building, realizing the deep integration of mental health education.

## 4 Construction of the mental health education system for college students

### 4.1 Inter-module systematic integration and theoretical mechanisms

The six core modules are not isolated operational units, but rather form an interdependent and dynamically feedback-driven ecosystem. The logic of their systematic integration lies in the fact that the efficacy of any single module is closely intertwined with the status of other modules, which are integrated into an organic whole through two core theoretical pathways: the mediating pathway of “educators’ mental health literacy” and the moderating pathway of “resource allocation.”

Specifically, as a critical moderating variable, resource allocation serves as the initial driving force and boundary condition for the system’s initiation and sustainability. Sufficient and rational resource investment directly determines the depth and scope of training in Module 1 (Educator Capacity Building), the scale and professionalization level of Module 3 (Grassroots Team Development), and the allocation of professionals and response speed in Module 4 (Crisis Intervention). The abundance or scarcity of resources, analogous to nutrient supply in an ecosystem, fundamentally regulates the growth ceiling and stability level of the entire system.

On this basis, the output of Module 1—high-level educators’ mental health literacy—plays a core mediating and transformative role. Such literacy enables educators to more acutely conduct observation and identification in Module 2 (Dynamic Monitoring), more standardizedly engage in collaborative response in Module 4 (Hierarchical Intervention), more intentionally guide atmosphere building in Module 5 (Classroom Environment), and implement personalized plans in Module 6 (Specialized Support) with greater empathy and professionalism. Educators’ literacy acts as a key converter that transforms static system “design” into dynamic “practice.”

Meanwhile, there exist strong mutually reinforcing effects among the modules: the grassroots network established in Module 3 greatly expands the monitoring touchpoints of Module 2; the positive environment fostered in Module 5 can significantly reduce the incidence of crisis events requiring intervention in Module 4; and the successful practice of Module 6 feeds back into Module 5, promoting a more inclusive classroom culture. Ultimately, the effective operation of the entire system will yield core outcomes of enhancing students’ mental well-being and facilitating their academic success. The institutional recognition and resource feedback brought by these positive outcomes will further consolidate and optimize the initial resource allocation, thereby forming a virtuous cycle aimed at continuously improving system efficacy. Thus, this framework not only describes static components but also uncovers the mechanisms of their internal dynamic interactions, providing a clear theoretical model for empirical testing.

### 4.2 Detailed introduction to the six core modules of the college students’ mental health education system

#### 4.2.1 Educator mental health capacity building module

Higher education educators serve as the core force in safeguarding teaching quality, deepening educational effectiveness, and maintaining campus operational efficiency (Zhang and Li, 2025). While their mental health status is not directly visible in work processes, it sustainably and profoundly influences the trajectory of educational outcomes. Positive and stable mental states can effectively enhance classroom appeal, optimize the quality of teacher-student interactions, and help foster a supportive and growth-oriented overall educational ecosystem. Conversely, long-term suboptimal or poor mental health (e.g., persistent anxiety, occupational burnout) is prone to triggering a “spillover effect” of negative emotions—this not only erodes teacher-student trust and hinders student development but also exerts subtle yet pernicious negative impacts on the campus’s overall psychological climate and educational atmosphere (Wu and Lu, 2022).

Constructing a systematic capacity-building module integrating self-regulation and professional support constitutes the key to enhancing educators’ psychological resilience. Grounded in self-regulatory strategies, this module includes daily focused breathing exercises and other mindfulness meditation practices, which help reduce distraction, enhance self-awareness, and alleviate cognitive fatigue induced by multitasking (Dave et al., 2020). Simultaneously, physical exercise—such as aerobic activities to relieve anxiety and strength training to foster a sense of physical mastery—provides diverse physiological and psychological regulatory pathways for stress management (Nonato et al., 2025). Collectively, these self-regulation methods form the foundational layer of the module, laying the groundwork for sustainable self-care capabilities that underpin subsequent in-depth professional support and interventions.

When psychological warning signs persist among higher education educators, a cognitive shift from “reactive coping” to “proactive care” should be promoted. When facing symptoms such as persistent low mood, sleep disturbances, or significant declines in work efficiency that last for more than 2 weeks, educators need to proactively overcome the stigma associated with seeking help, framing professional support-seeking as a manifestation of proactive self-care and professional responsibility (Agyapong et al., 2022). Through university mental health centers and university-enterprise collaborative professional support platforms, they can access systematic psychological assessments and targeted interventions to effectively address stressors and emotional exhaustion, thereby achieving positive recovery of psychological well-being.

In summary, the synergistic module of self-regulation and professional support enables preventive daily management and early crisis intervention. This module enhances educators’ psychological resilience, serving as an indispensable underpinning for the stable and efficient operation of university education systems.

#### 4.2.2 Student psychological state dynamic monitoring module

Amid the complex and volatile social environment and the frequent occurrence of college students’ mental health issues, accurately grasping students’ mental states has emerged as a paramount prerequisite for universities to conduct mental health education. College students are in a critical period of physical and psychological development, vulnerable to the impacts of multiple factors such as academic pressure, interpersonal relationships, employment anxiety, and family environments, thus exhibiting dynamic changes in their mental states. Failure to promptly detect such psychological fluctuations may lead to the deterioration of mental health problems and even trigger extreme incidents. Therefore, organically integrating regular mental health screenings, professional data interpretation, and multi-channel dynamic observation to construct a comprehensive, multi-level early warning network for students’ mental health constitutes the top priority of university mental health work. The core of this early warning network lies in relying on “multi-role collaborative entities” to form a synergetic work force. For student groups at different developmental stages (e.g., freshmen, academically challenged students, graduates, and special groups such as students from low-income families), establishing a closed-loop system of “regular screening—professional decoding—dynamic tracking” is a key foundation for achieving “early identification and early intervention” of psychological crises.

The effectiveness of routine psychological screening depends on the scientific selection of screening timing and the appropriate application of assessment tools. Implementing universal screening during the critical three-month adaptation period following freshmen enrollment facilitates the timely identification of common issues such as adjustment disorders. At this stage, the Symptom Checklist-90 (SCL-90; Derogatis et al., 1973) can be adopted for multidimensional assessment of freshmen’s psychological states, as this scale effectively reflects core indicators including interpersonal sensitivity, obsessive-compulsive tendencies, and anxiety levels (Cao, 2022; Sugawara et al., 2023). For graduates in the job-seeking phase, supplementing a specialized employment anxiety scale prior to the employment season enables accurate capture of this group’s specific psychological states and needs related to career planning, competitive pressure, and other aspects, thereby providing a basis for delivering phased and targeted psychological support.

Professional interpretation of screening data constitutes the core component for enhancing the accuracy of risk assessment and avoiding misjudgment, as mental health professionals at university counseling centers should conduct hierarchical analysis of collected data, preliminarily categorizing students’ psychological states into three tiers—no-risk, low-risk, and moderate-to-high-risk—based on scores from standardized scales and generating structured assessment reports for each tier; a critical point, however, is that this interpretation process should not be confined to quantitative scores but must systematically integrate qualitative information such as students’ family backgrounds, significant life events, and academic performance to enable contextualized understanding of risks and mitigate classification bias arising from overreliance on numerical data alone, and without this “assessment-interpretation loop,” two key issues are likely to emerge: first, data underutilization, where screening results remain merely statistical and fail to be effectively translated into a basis for early warning and intervention; second, disconnected monitoring, characterized by insufficient dynamic tracking of high-risk groups or potential issues that results in missing critical opportunities for early prevention and intervention.

Multi-channel monitoring synergy and differentiated adaptation further enhance the network’s inclusiveness and accuracy. As core implementers, counselors foster a relaxed and trusting communication atmosphere to empathetically listen to students’ academic, daily, and interpersonal difficulties, capturing psychological signals embedded in verbal expressions, emotional fluctuations, and abnormal behaviors; they also conduct continuous daily observation of attendance, dormitory dynamics, and social participation to promptly identify potential risks. Specialized course teachers leverage teaching scenario advantages by using classroom psychological behavior observation checklists to track key indicators including attention concentration, classroom interaction frequency, and homework submission quality, with timely feedback of abnormal changes to counselors or mental health centers. Student cadres, as “frontline informants” with close daily peer interactions, play a crucial supplementary role: universities incorporate them into a specialized training system covering mental health literacy (to understand common psychological problems and manifestations), abnormal behavior identification skills (to enhance detection capabilities), and standardized reporting procedures (to clarify timely feedback to counselors when signs such as sleep disorders, social withdrawal, or persistent low mood are observed in peers), ensuring the timeliness and accuracy of information transmission.

In summary, constructing a scientific and comprehensive dynamic monitoring and early warning network for students’ mental health constitutes an indispensable support for safeguarding college students’ mental well-being and optimizing the university educational ecology. Universities should continuously improve the monitoring system, strengthen multi-role collaboration, enhance the accuracy of monitoring and intervention, and safeguard the healthy growth of college students.

#### 4.2.3 Grassroots psychological service team building module

Currently, higher education institutions generally face the practical predicament of relatively limited mental health service resources. Specifically, academic advisors are typically responsible for guiding 150 to 200 students, posing objective challenges to their ability to conduct in-depth and comprehensive monitoring of individual students’ psychological dynamics. Relying solely on the academic advisor team makes it difficult to achieve systematic monitoring and early identification of students’ mental health status; as a result, potential psychological distress among some students may be overlooked, delaying the delivery of necessary support and intervention. Against this backdrop, constructing a systematic and hierarchical grassroots mental health support network has become particularly urgent. Establishing a three-tiered peer support system—with class psychological committee members as key nodes, Student Party members and backbone students as the core force, and enthusiastic students at large as the participation base—has emerged as a crucial practical pathway to remedy the insufficiency of professional service coverage and extend the reach of mental health education to students’ daily academic and living scenarios. This model aims to effectively expand the coverage and accessibility of psychological support through natural peer attention, preliminary listening, and resource linkage, laying a foundation for early identification and intervention, thereby enhancing the overall effectiveness and response speed of the university’s mental health service system (Pointon-Haas et al., 2023).

The core advantage of this three-tiered peer support framework lies in its systematic division of roles and functional complementarity, which fosters a peer service system with clear hierarchies and collaborative operation. As the core nodes of the framework, class psychological committee members leverage their strengths of high-frequency and close-range peer interactions to undertake foundational support functions, including daily observation of psychological states, preliminary emotional comfort, and identification and reporting of crisis signals. In natural contexts such as dormitory communications and classroom collaborations, they can acutely detect subtle emotional and behavioral changes in peers that are not easily apparent, and provide preliminary empathic listening and emotional support to students experiencing mild psychological distress. As the backbone force, Student Party members exert dual roles of “role modeling and resource linkage” by virtue of their strong sense of responsibility, organizational coordination capabilities, and credibility among peers. On one hand, they actively participate in and advocate for mental health activities, helping to foster a positive and open class psychological climate; on the other hand, when peers are in need, they assist them in understanding and accessing formal campus mental health resources (e.g., booking individual counseling sessions, participating in group counseling), serving as a bridge between the peer support system and professional service systems. As the broad participation layer, students passionate about mental health services enrich the presentation forms and coverage of mental health education primarily through participating in thematic activity planning, promotional material development, and popular science dissemination. Their involvement helps cultivate a collective identity within the class where “mental health is everyone’s concern and mutual support is prevalent,” thereby enhancing the overall effectiveness of early prevention.

The in-depth integration of a systematic training system and an institutionalized collaboration mechanism is crucial to consolidating team effectiveness (Bohnenkamp et al., 2023). In terms of training system construction, a training mechanism featuring “full coverage, professionalization, and regularization” should be established. Specialized training for class psychological committee members is conducted regularly each year, with the content focusing on three core modules: Knowledge Popularization Module: Systematically teaches basic theories of mental health, as well as the causes, typical manifestations, and identification methods of common mental health issues such as depression and anxiety (Nobre et al., 2021). These drills enable psychological committee members to master core communication skills such as active listening, empathy, and open-ended questioning in simulated contexts, enhancing their practical counseling capabilities. Crisis Management Module: Clarifies the standardized process of “identifying abnormal signals—promptly reporting to counselors—assisting with preliminary comfort.” Key emergency response points are analyzed through real psychological crisis cases, such as how to remain calm, avoid provoking the individual, and simultaneously contact counselors when a peer expresses suicidal ideation. Post-training assessment adopts a two-dimensional approach combining “knowledge tests + practical skill evaluations” to ensure a 100% pass rate for psychological committee members. Additionally, psychological committee members are encouraged to regularly conduct “micro-classes on mental health knowledge for the class,” extending the coverage of mental health education to all classmates.

This effectively fills the gap in professional mental health service resources in universities. Meanwhile, the team serves as a core pillar for building an “all-staff, whole-process, and all-round” mental health education system, providing solid grassroots support for universities to shift from “passive response” to “proactive prevention” in mental health services and helping foster a positive campus mental health environment.

#### 4.2.4 Hierarchical psychological crisis intervention module

The hierarchical psychological crisis intervention and management system for college students serves as a critical component and core emergency response mechanism of the campus mental health protection system. Designed around a scientific and structured response framework, this system aims to achieve three key objectives: precision risk identification, rapid hierarchical response, and systematic efficient management. Specifically, it enables timely intervention during the initial stage of a crisis to prevent deterioration into extreme incidents; throughout the crisis management process, it provides professional and humanistic systematic support to assist students in restoring psychological balance and rebuilding adaptive capacities. The effective operation of this system relies on the organic integration of three pillars: the grassroots support network, standardized hierarchical intervention procedures, and multi-departmental collaborative linkage. The grassroots support network forms the foundation for early crisis identification and reporting; hierarchical intervention ensures that crises of varying severity receive proportionate professional responses; and cross-departmental collaboration guarantees the efficiency of resource integration and unified action in major crises. Together, these pillars form a three-dimensional protection and support closed loop that covers the entire crisis lifecycle and connects all levels of the campus.

Grassroots support serves as the foundation for the effective operation of this module, with its core goal of achieving “early detection and early warning” of psychological crises; to this end, a systematic defense line should be constructed from two dimensions: preventive publicity and education, and screening and file establishment. In terms of preventive publicity and education, university mental health centers should take the lead in collaborating with various departments and schools to establish a “phased and focused” publicity and education system, carrying out differentiated activities targeting the psychological development characteristics and common predicaments of students in different grades: for freshmen who have just entered the campus and face adaptation and interpersonal challenges, thematic lectures on “Campus Adaptation and Interpersonal Boundaries” are organized, using case analyzes and other forms to help them quickly integrate into the campus and master communication skills; for sophomores and juniors with concentrated academic pressure and significant emotional fluctuations, psychodrama performances can be carried out, focusing on themes such as “Academic Anxiety Regulation,” “Emotional Conflict Resolution,” and “Self-Worth Exploration,” encouraging students to write and perform real campus stories themselves, and learn psychological adjustment methods through experience and observation; for graduates facing dual pressures of employment and future planning, special activities on “Job Search Anxiety Relief” can be designed, such as the essay competition “My Job Search Story” and the painting creation “Emotional Colors,” guiding them to release emotional pressure through written and artistic expression.

“Hierarchical intervention and cross-departmental collaboration” serve as the core for enhancing the efficiency of psychological crisis disposal, whose effective operation relies on clear crisis classification criteria, corresponding responsible entities for intervention, and specified response timeframes. As key implementers of this system, educators—with counselors as the primary force and class teachers as supplements—must continuously strengthen their core competencies (Nielsen et al., 2024). First and foremost, educators should systematically master the “four-step crisis identification framework”: Step 1, observe emotional manifestations (e.g., persistent depression, irritability, or sudden emotional outbursts); Step 2, assess behavioral changes, including disrupted sleep/eating patterns, social withdrawal, or significant declines in academic performance; Step 3, evaluate social support resources to confirm whether students have reliable emotional support from family, peers, or teachers; Step 4, integrate the aforementioned information to determine risk levels, providing a basis for subsequent interventions. Furthermore, educators should conduct regular in-depth interviews with moderate-to-high-risk students included in the database to dynamically track changes in their psychological states.

In the implementation phase of hierarchical intervention, differentiated strategies should be adopted based on the severity of the crisis: For mild psychological distress—such as short-term emotional fluctuations, minor interpersonal conflicts, or temporary academic pressure—immediate support channels (e.g., on-campus psychological counseling hotlines and online platforms) should be activated; counselors may simultaneously provide short-term empathetic listening and problem-solving guidance to help students alleviate emotional stress. For persistent neuropsychological symptoms (e.g., depression or anxiety lasting more than 2 weeks), full-time psychological counselors should take proactive intervention, employing professional methods such as cognitive-behavioral therapy (CBT) and mindfulness-based stress reduction (MBSR) for systematic intervention, while regularly evaluating progress and adjusting treatment plans. In the event of severe crises—including suicidal ideation, suicidal behaviors, or acute mental disorder episodes—a cross-departmental emergency response mechanism must be activated immediately, with a collaborative team consisting of psychological counselors, university doctors, academic advisors, and parents intervening promptly: University doctors prioritize physical health screening, counselors take charge of emotional stabilization and safety maintenance, and communication with parents is initiated immediately to assist in referring students to professional mental health institutions, ensuring timely access to specialized treatment and minimizing the consequences of the crisis.

This module achieves accurate and efficient disposal of crises of varying severity through “pre-positioned grassroots defense lines, precise hierarchical intervention, and cross-departmental collaborative guarantees.”

#### 4.2.5 Mental health-friendly class environment co-construction module

As a key venue for academic exchange and emotional support among college students, the class climate exerts a profound impact on students’ mental health through three interrelated mechanisms—"emotional bonding, identity recognition, and stress buffering” (Hoferichter et al., 2022). Positive emotional bonds enhance a sense of belonging and security, thereby alleviating feelings of loneliness; a shared class identity fosters self-worth and psychological resilience; and peer support effectively mitigates the adverse effects of academic pressure and interpersonal conflicts. However, prevalent characteristics in contemporary university education—such as cross-campus course selection, fragmented curriculum scheduling, and insufficient participation in collective activities—have objectively undermined sustained and in-depth interpersonal interaction and emotional communication among class members, easily leading to a class climate characterized by “superficial familiarity yet emotional estrangement.” Such prolonged estrangement leaves students unable to access timely and effective social support when confronting setbacks, hindering the regulation of negative emotions and potentially exacerbating psychological issues such as anxiety and depression. Consequently, proactively constructing a positive class environment centered on the principles of “strengthening emotional bonds through activities and regulating class order via systems,” while seamlessly integrating mental health education into grassroots class construction, has become a crucial practical direction for universities to safeguard students’ mental health.

To address the current lack of class cohesion, constructing a hierarchical activity system that is precisely aligned with students’ psychological development stages and features a “phased and all-encompassing” design constitutes an effective approach to strengthening the interpersonal support network within classes. This system is tailored specifically to the key developmental tasks and needs of students in different grades: During the freshmen adaptation period, within 3 months of enrollment, immersive ice-breaking activities are intensively organized—such as team puzzle challenges integrated with class cultural elements and class narrative sharing sessions—aimed at promoting familiarity and trust among members through structured collaboration and in-depth communication, thereby alleviating initial adaptation anxiety (Fjermestad et al., 2025). For sophomores and juniors, in response to concentrated academic pressure and significant emotional fluctuations, experience-sharing workshops themed around “Academic Stress Management and Emotional Regulation” are held; top-performing senior students are invited to share learning strategies, and peer pressure coping discussions are conducted to guide students in proactively addressing academic and emotional challenges. During the graduation transition period, the focus shifts to employment anxiety and role transition, with a series of activities including alumni experience sharing sessions, interview skills workshops, and career planning counseling; through experiential activities such as “future career role simulation,” students are helped to alleviate identity confusion and enhance employment confidence. All thematic activities are designed based on the hierarchical psychological needs theory, ensuring the pertinence and systematicity of the intervention (Viner et al., 2025).

To ensure that the mental health-oriented class environment can sustainably and stably fulfill its supportive role, in addition to emotion-building activities, establishing a class management system characterized by clear division of responsibilities, procedural justice, and appropriate flexibility is crucial (Gao, 2025). This system should strike a balance between standardized operations and students’ autonomous participation to foster an equal and inclusive class climate. In terms of teacher-student interaction and class decision-making, counselors can regularly solicit opinions through online questionnaire platforms (e.g., Wenjuanxing, a widely used Chinese survey tool) and offline student representative forums. Reasonable suggestions related to class management, activity planning, and psychological support should be timely responded to and implemented, forming a democratic mechanism of two-way communication. The selection of class committee members must follow open and transparent democratic procedures: clarify job responsibilities and candidacy requirements prior to the election, allow candidates to present their work plans through public statements, and finally elect members via anonymous voting—this enhances students’ sense of identification and reduces potential conflicts thereafter. Within the class committee, a collaborative division of labor with complementary functions should be established: psychological committee members regularly report the class’s emotional dynamics and potential risks, academic committee members organize academic support groups, and life committee members coordinate dormitory relationships and daily affairs, thereby constructing a multi-dimensional and collaborative class support network (Besse et al., 2024). The design of the class reward and punishment system should balance guiding and supportive functions. For students who actively participate in mental health promotion activities and proactively care for peers, positive incentives such as public recognition, certificates of merit, or extra points in comprehensive evaluations can be implemented. For disciplinary behaviors such as being late or absent, a hierarchical handling process of “identifying causes → reflecting on plans → supporting improvement” should be adopted: clarify the root cause of the problem through one-on-one interviews, guide students to formulate written improvement plans, and arrange peers or class committee members to provide ongoing attention and support during the process. This approach maintains the seriousness of the system while providing students with room for behavioral correction and growth recovery (Ijaz et al., 2024).

The synergistic integration of “emotional connection through activities and order regulation through systems” effectively addresses interpersonal alienation in university classes, strengthens mental health support networks, and consolidates class harmony through institutional guarantees. This co-construction module enables the class to function as a fertile ground for nurturing mental health, playing an irreplaceable role in stabilizing the campus mental health ecosystem and promoting students’ holistic well-being.

#### 4.2.6 Targeted support module for special student groups

Special student groups in higher education often face more complex growth challenges than their peers due to objective disparities in economic conditions, physical and mental status, and academic foundations (Solís García et al., 2024; Vicary et al., 2025). Specifically, students from low-income backgrounds are prone to inferiority and social anxiety stemming from economic gaps; students with mental health concerns must constantly balance symptom management, academic persistence, and daily life; academically challenged students frequently experience low self-efficacy and academic burnout due to lagging academic performance; and students with disabilities may encounter multiple barriers to learning methods and social integration as a result of physical or sensory impairments. The psychological states of these groups are often more vulnerable—without targeted and systematic support, not only will their practical difficulties remain unaddressed, but their psychological stress may also be exacerbated, leading to a “cumulative adversity effect.” Therefore, constructing a multi-dimensional support system centered on “precision identification, classified support, and collaborative guarantee” is not only an inevitable choice to address the differentiated needs of special student groups but also a key pathway to advancing college mental health education toward refinement, personalization, and ecologicalization. This system aims to translate the concept of “all-staff education, whole-process education, and all-round education” into a concrete and operable practical framework. Through institutionalized and professional support mechanisms, it effectively enhances the psychological resilience, academic adaptation, and overall well-being of special student groups, thereby fostering a more inclusive and equitable educational environment.

Precision identification serves as the foundation for constructing an effective support system, requiring a multi-path assessment mechanism integrating “psychological screening, in-depth interviews, and daily observation” to comprehensively grasp students’ conditions, avoid information omissions, and thereby provide a reliable basis for subsequent interventions (Garcia et al., 2024; Gull et al., 2025; Vicary et al., 2025). In the psychological screening phase, standardized tools with good reliability and validity should be adopted to focus on assessing core psychological indicators of different special student groups; risk stratification is conducted based on Chinese norms to initially identify moderate-to-high-risk individuals. Simultaneously, one-on-one in-depth interviews must be conducted to collect qualitative information, which supplements and corrects quantitative screening results—for instance, distinguishing between temporary anxiety among students from low-income families caused by short-term financial pressure and persistent emotional disorders in students with mental health needs, thus preventing assessment biases arising from over-reliance on numerical scores. In-depth interviews should involve targeted dialogs centered on the key predicaments of different groups: When communicating with students from low-income families, it is necessary to understand the true details of their family economic status under the premise of privacy protection, while paying attention to the impact of financial pressure on their self-worth and social confidence; when engaging with students with mental health needs, the focus should be on exploring symptom changes, medication adherence, social functioning maintenance, and potential crisis signals; when conversing with academically challenged students, it is essential to analyze the causes of academic difficulties while assessing whether they are accompanied by psychological states such as academic burnout, anxiety, or self-denial; When interacting with students with disabilities, emphasis should be placed on understanding the specific barriers and support needs they face in learning environments and social participation. Furthermore, a systematic daily observation mechanism should be established with differentiated observation checklists: For academically challenged students, record their classroom engagement, homework completion, and changes in academic performance; for students with disabilities, monitor their participation in classroom and extracurricular activities as well as the quality of peer interactions; for students from low-income families, observe their consumption patterns, social withdrawal behaviors, and sensitive responses to financial topics; for students with mental health needs, track their emotional stability, regularity of daily routines, and changes in social interactions. By integrating the aforementioned multi-dimensional and multi-source information, a holistic understanding of students’ psychosocial conditions can be formed, laying the foundation for the subsequent development of personalized support plans.

To deliver effective support for special student groups, the principle of “one student, one plan” should be adhered to, and a demand-oriented personalized support system constructed to ensure that interventions are precisely aligned with students’ actual predicaments. For students from low-income families, a dual-track support model integrating financial assistance and psychological empowerment is adopted. At the financial level, systematic linkages are established with resources such as national grants and on-campus work-study positions; for eligible students (e.g., orphans and those from registered poor families), procedures for tuition reduction/exemption and temporary hardship subsidies are streamlined to effectively alleviate subsistence pressure. At the psychological level, semesterly growth education themed “Healthy Consumption Values and Self-Worth Recognition” is conducted. Through case discussions, cognitive-behavioral training, and other methods, students are guided to break free from the cognitive constraint that “economic status determines self-worth,” mitigate anxiety and self-denial stemming from material disparities, and gradually develop a positive, holistic self-identity. For students with mental health needs, a tripartite collaborative follow-up support mechanism involving university doctors, psychological counselors, and academic advisors is established. University doctors monitor medication adherence and physical responses regularly (e.g., monthly); psychological counselors provide weekly cognitive-behavioral interventions to help students adjust irrational beliefs and enhance emotional regulation skills; academic advisors track emotional changes and social adaptation through daily communication, forming a closed-loop support system encompassing medical care, psychological support, and daily management. For academically challenged students, an academic improvement system combining personalized mentoring by faculty advisors and peer support groups is developed. Faculty advisors diagnose academic difficulties, formulate tailored learning plans, and impart evidence-based study strategies; peer support groups consisting of high-achieving students offer regular academic tutoring and emotional encouragement. Simultaneously, consistent communication is maintained with parents to guide them in replacing criticism with encouragement, collectively fostering an environment conducive to students rebuilding academic confidence. For students with disabilities, barrier-free support balancing learning condition adaptations and social integration assistance is provided. In academic settings, reasonable accommodations are tailored to the type and severity of disabilities—for example, real-time subtitles or sign language interpreters for deaf or hard-of-hearing students. For social integration, students are supported to leverage their strengths and build self-confidence through initiatives such as art performance participation and low-pressure social activities, gradually enhancing social engagement and self-efficacy within an inclusive environment (Minotti et al., 2021).

In summary, the multi-dimensional support system for special student groups thoroughly addresses the drawbacks of traditional one-size-fits-all support and “form-over-substance” practices through “precision identification to eliminate blind spots, classified measures to meet differentiated needs, and collaborative mechanisms to ensure effectiveness.”

## 5 Discussion

### 5.1 Review findings and framework significance

This review systematically synthesizes existing evidence and proposes a comprehensive support framework for college student mental health education consisting of six core modules. The framework advances theoretical development and practical transformation in the field along three dimensions:

First, theoretical integration and systematic construction: Beyond the isolated evaluation of individual interventions, the framework provides a logically consistent and fully fledged systematic structure. It organically integrates fragmented practical components such as curriculum development, monitoring, intervention, and environment building, depicting a multi-level and dynamically interconnected mental health education ecosystem. This offers a holistic perspective for understanding and designing campus mental health initiatives.

Second, mechanism clarification and testable pathways: The framework explicitly identifies two actionable and testable core theoretical mechanisms. The mediating role of educators’ mental health literacy clarifies how system resources and policy inputs are translated into high-quality student support practices by enhancing educators’ knowledge, skills, and self-efficacy, uncovering the key transformation pathway from “system design” to “student benefit.” The moderating effect of resource allocation specifies how institutional-level resource conditions (e.g., human resources, financial resources, and institutional support) act as critical boundary conditions to regulate the implementation depth, coverage scope, and ultimate effectiveness of each module within the framework, providing a theoretical basis for explaining the variability in implementation outcomes across different institutions.

Third, practice orientation and application flexibility: Far from an abstract model, all six modules of the framework—educator capacity building, dynamic monitoring, grassroots teams, hierarchical intervention, environment co-construction, and targeted support—correspond to identifiable and actionable practice areas. This provides universities with a structured tool to assess their current work foundations, identify gaps, and plan development pathways. It supports institutions in gradually constructing and refining a mental health system characterized by “whole-staff participation, whole-process integration, and whole-campus coverage,” reflecting application flexibility that bridges universal principles with school-based practice, tailored to their unique resource endowments and cultural contexts.

### 5.2 Current research gaps and future directions

Although this framework has received scattered support from existing literature, there are significant gaps in current research that urgently require filling through future empirical work: First, the lack of testing on overall system effectiveness—most studies evaluate individual programs or measures, and there is almost no research assessing the long-term overall effectiveness of such a multi-module composite ecosystem. Second, insufficient validation of core mechanisms: there is a dearth of longitudinal or experimental research evidence regarding how “educators’ mental health literacy” specifically influences students through its mediating role, and how different models of “resource allocation” moderate the effectiveness of each module. Third, a gap in cross-cultural adaptability research: elements such as “student Party member backbones” and “counselor-centered system” in the framework are culturally specific to China, and their applicability and adaptation approaches under other cultural and educational systems call for in-depth comparative research.

### 5.3 Limitations

The limitations of this review lie in its scope-related nature: it did not conduct rigorous quality assessment of the included literature, and the framework construction contains a certain degree of deductive elements.

## 6 Conclusion

Faced with the complex and systematic challenges of college students’ mental health issues, piecemeal responses have proven inadequate. The comprehensive support system framework comprising six core modules integrated and proposed in this review provides a holistic conceptual guidance for universities to establish a mental health work system characterized by prevention orientation, whole-staff participation, rapid response, and precise support. Going forward, researchers and practitioners should build on this framework to conduct rigorous empirical research—particularly validating its internal mediating and moderating mechanisms—and implement local adaptation and innovation in diverse contexts, thereby truly advancing college students’ mental health education into a new phase of systematic science.

## Data Availability

The original contributions presented in the study are included in the article/supplementary material, further inquiries can be directed to the corresponding author.
